# Differences in prefrontal cortex activation and deactivation during strategic episodic verbal memory encoding in mild cognitive impairment

**DOI:** 10.3389/fnagi.2015.00147

**Published:** 2015-08-04

**Authors:** Joana B. Balardin, Marcelo C. Batistuzzo, Maria da Graça Moraes Martin, João R. Sato, Jerusa Smid, Claudia Porto, Cary R. Savage, Ricardo Nitrini, Edson Amaro, Eliane C. Miotto

**Affiliations:** ^1^Departamento de Radiologia, Faculdade de Medicina, Universidade de São PauloSão Paulo, Brazil; ^2^Departamento de Neurologia, Faculdade de Medicina, Universidade de São PauloSão Paulo, Brazil; ^3^Centro de Matemática, Computação e Cognição, Universidade Federal do ABCSanto André, Brazil; ^4^Department of Psychiatry and Behavioral Sciences, Center for Health Behavior Neuroscience, University of KansasKansas City, KS, USA

**Keywords:** mild cognitive impairment, age-related memory disorders, verbal episodic memory, fMRI, semantic encoding

## Abstract

In this study we examined differences in fMRI activation and deactivation patterns during episodic verbal memory encoding between individuals with MCI (*n* = 18) and healthy controls (HCs) (*n* = 17). Participants were scanned in two different sessions during the application of self-initiated or directed instructions to apply semantic strategies at encoding of word lists. MCI participants showed reduced free recall scores when using self-initiated encoding strategies that were increased to baseline controls' level after directed instructions were provided. During directed strategic encoding, greater recruitment of frontoparietal regions was observed in both MCI and control groups; group differences between sessions were observed in the ventromedial prefrontal cortex and the right superior frontal gyrus. This study provides evidence suggesting that differences of activity in these regions may be related to encoding deficits in MCI, possibly mediating executive functions during task performance.

## Introduction

Mild cognitive impairment (MCI) is a heterogeneous syndrome that in some cases is transitional between normal age-related cognitive changes and dementia (Patel and Holland, 2012). MCI with episodic memory (EM) impairment, namely amnestic MCI (aMCI), has been identified as a possible precursor of Alzheimer Disease (AD) (Petersen et al., 2001; Albert et al., 2011). Impairments in EM in aMCI and early AD patients can be identified by reduced performance in delayed free recall measures, such as word list-learning tasks (Jak et al., 2009). In AD patients, this impairment is often attributed to medial temporal lobe (MTL) neuropathology. In MCI, however, the exact nature of the verbal episodic memory deficit is unknown. It has been proposed that it can result not only from deficits in acquisition and consolidation processes characteristic of AD, but also from attentional or executive functions deficits that lead to inefficient encoding and/or retrieval of verbal material (Twamley et al., 2006).

Prior fMRI studies of memory in aMCI have produced mixed results of decreased and increased hippocampal activation that seems to result from the large variability between studies in disease classification and severity as well as in the characteristics of the memory tasks (for a review see Dickerson and Sperling, 2008). Specifically, studies examining verbal memory encoding processes have also reported inconsistent activation patterns in frontoparietal regions in MCI relative to controls (Hämäläinen et al., 2007; Dannhauser et al., 2008; Clément and Belleville, 2010). There is also evidence suggesting that these group differences may be modulated by task characteristics, since individuals with MCI and clinical AD exhibited less suppression of the so-called default mode network regions than healthy older adults in response to increases in task demands during, for example, working memory encoding (Lustig et al., 2003; Buckner et al., 2008; Kochan et al., 2010).

Despite the above contributions to the identification of the neural substrates underlying memory impairment in MCI, an aspect still not investigated is the contribution of the cognitive strategies adopted by MCI subjects to perform episodic verbal learning tasks. It has been demonstrated that older adults are not as able as young subjects in using spontaneous verbal learning strategies during word-list learning tasks in order to improve episodic memory recall (Fernandes and Grady, 2008), and that this pattern tends to deteriorate along the MCI-AD continuum (Ribeiro et al., 2007; Hutchens et al., 2012). At least in cognitively healthy older adults, the provision of semantic elaboration strategies during intentional verbal encoding was shown to be effective in improving memory performance and inducing increases in ventrolateral PFC activation, a region that was initially under-recruited (i.e., decreased fMRI activation) in comparison to young adults (Logan et al., 2002). However, it is unknown whether similar cognitive mechanisms would operate in the presence of episodic learning impairment that may be accompanied by subclinical neuropathology in memory-related regions, such as in MCI. Findings from memory strategic-training studies in MCI show that increased activation of prefrontal, temporal, and parietal regions were associated with improved memory performance for word-lists (Belleville et al., 2011) and face-name associations (Hampstead et al., 2011), suggesting a possible malleability of changes in cognitive and neural processing in this population.

In the present study we therefore investigated the neural correlates of differences in verbal learning strategy application during episodic memory encoding in MCI and age-matched healthy controls (HCs). Different levels of strategic processing were manipulated during unconstrained intentional encoding of word lists and after an explicit, direct instruction to apply a semantic encoding organizational strategy. We predicted that, relative to the unconstrained intentional encoding condition, both control and MCI groups would exhibit increases in memory and strategic performance after the explicit orientation to apply the semantic organizational strategy that would be paralleled by increased recruitment of frontoparietal network regions during episodic verbal encoding. We also investigated whether the MCI group would exhibit patterns of overactivation in frontoparietal network regions and/or impaired suppression (i.e., less deactivation) in DMN regions compared to controls during verbal episodic encoding after the explicit orientation to apply the semantic learning strategy.

## Materials and methods

### Participants

A total of 18 MCI patients and 17 HCs, all right handed, were included in the study (demographic and neuropsychological profile are given in Table 1). The MCI patients were recruited from a specialized Alzheimer's disease clinic (CEREDIC) and in the Behavioral Neurology section at the Hospital das Clínicas, University of Sao Paulo, Sao Paulo, Brazil. Patients with MCI were diagnosed using the criteria suggested by Petersen (Petersen et al., 2001), which was operationalized in our study as the following: presence of memory complaint corroborated by an informant, performance of at least 1 SD below the mean adjusted by age on the Rey Auditory Verbal Learning Test (RAVLT) adapted to the Brazilian elderly population (Malloy-Diniz et al., 2007), normal general cognitive function assessed by Mini-Mental State Examination (adjusted for age and education) (Brucki et al., 2003) and no impairment in activities of daily living. Given our interest in including individuals at the very early stages of the MCI spectrum, the threshold for determining memory impairment was 1 SD below the age norms instead of the more conventional criteria of 1.5 SD, on the delayed recall of the RAVLT. In addition, to be included in the study, each participant had to receive a consensus diagnosis (Winblad et al., 2004) incorporating clinical history, medical records, laboratory evaluation, and neuroimaging exams by an evaluating physician and a neuropsychologist from the team. The HCs were independently functioning members of the community and did not meet MCI criteria. Exclusion criteria included presence of intracranial lesion detected in the structural MRI visually checked by a neuroradiologist (i.e., evidence of ischemic or hemorrhagic stroke or space-occupying lesions; small foci of T2 hyperintensities were not excluded, but were classified qualitatively), any type of dementia or any other type of disease that might impair cognitive function (e.g., depression), and current or past alcohol or drug abuse. Participants were excluded also on factors based on MRI contraindications such as metallic implants and claustrophobia. All subjects had normal vision or that corrected to the normal standard by the use of MRI-compatible eyeglasses. The study was approved by the local ethics committee (CAPPesq *0349/09*) and the patients signed a written informed consent form prior to their inclusion in the study.

**Table 1:** **Characteristics of the subjects with MCI and controls**.

	**Controls (*n* = 17)**	**MCI (*n* = 18)**	***p*-values**
Age	68.25 (1.54)	69.50 (1.91)	0.652
Education	11.19 (1.35)	9.20 (1.13)	0.332
Sex	8M/9F	8M/10F	0.870
Fazekas score—DWM	1.21	1.5	0.107
Fazekas score—PVWM	1.38	1.15	0.375
GDS	1.25 (0.38)	1.69 (0.41)	0.444
Pfeffer	0.27 (0.2)	2.19 (0.52)	0.002
MMSE	28.33 (0.37)	27.06 (0.53)	0.167
Paragraph immediate recall (WMS-R)	26.36 (1.60)	20.61 (2.10)	0.053
Paragraph delayed recall (WMS-R)	24.36 (1.44)	9.06 (1.59)	< 0.001
RAVLT immediate total recall	46.60 (2.89)	27.94 (2.22)	< 0.001
RAVLT immediate recall	9.80 (0.67)	5.24 (0.59)	< 0.001
RAVLT delayed recall	9.44 (0.65)	4.41 (0.46)	< 0.001
Digit span forward (WAIS)	7.17 (0.52)	5.06 (0.34)	0.002
Digit span backward (WAIS)	5.42 (0.41)	3.06 (0.14)	< 0.001
Rey figure (copy)	31.50 (1.24)	30.69 (1.22)	0.649
Rey figure (recall)	14.62 (1.78)	8.63 (1.71)	0.024
Stroop (time on third plate)	31.77 (2.28)	43.39 (5.31)	0.045
Verbal fluency (supermarket, MDRS)	24.64 (2.53)	17.79 (1.40)	0.020
Verbal fluency (FAS)	38.33 (2.61)	27.24 (3.42)	0.024
Boston naming	54.07 (1.20)	48.83 (2.18)	0.020

*Results are expressed as mean (SE)*.

*DWM, deep white matter; PVWM, peri-ventricular white matter; GDS, Geriatric Depression Scale; MDRS, Mattis Dementia Rating Scale; MMSE, Mini-Mental State Examination; WMS-R, Wechsler Memory Scale Revised; WAIS, Wechsler Adult Intelligence Scale*.

### fMRI experimental paradigm and procedure

Participants were scanned in two fMRI sessions, being described here as spontaneous and directed encoding conditions, distinguished by an explicit guidance on how to apply semantic clustering during intentional verbal encoding. The fMRI word list learning paradigm consisted of alternating blocks of encoding and resting baseline conditions. The encoding blocks required subjects to read silently and intentionally memorize lists of concrete nouns visually presented on the screen for subsequent recall. Two different sets of lists were used. One included lists formed by 16 words grouped into four semantic categories of four words each (SR list, ex. fruits, musical instruments, vegetables, tools). The general approach to manipulating semantic organization was modeled closely on the CVLT, which is a well-characterized clinical measure of strategic verbal memory (Delis et al., 1998). The other set was formed by 16 words that were not semantically related to one another (UR list). Word lists were balanced for word length and their validity in prompting significant differences in semantic clustering was tested in previous studies (for a more detailed description, see Savage et al., 2001; Miotto et al., 2006). During the encoding blocks, each word was presented for 2.06 s. Words from the SR list were presented so that no two words from the same semantic category occurred consecutively, thus requiring active semantic and executive processing to clustering on subsequent free recall. Encoding blocks of each list condition were alternating with a 12 s resting baseline in which participants were oriented to fix the gaze into a cross in the center of the screen. Each word list block repeated three times through one run, in each fMRI session. To perform the spontaneous fMRI session, participants were not instructed about the semantic organization of the words in the lists beforehand or given any practice with related lists. Therefore, any grouping by category observed in the subsequent free recall at the end of this fMRI acquisition was presumed to be self-initiated by the subject. At the end of the spontaneous session, participants were taken to a different room and given a period of instructions and practice to apply semantic organizational strategies to a set of five different word lists. Subjects were equally instructed to organize the words into categories and to retrieve them according to their category. The practice period had occurred during a limited time (i.e., up to 30 min) until each participant was able to apply the categorization strategy to at least three different word lists. All participants were able to learn and apply the semantic strategies. Immediately after practicing the application of the strategy, participants were scanned again using the same type of paradigm as in the first session, except for the use of new set of word lists and the explicit instruction to apply semantic clustering. The presentation order of the words in each list was randomized and block conditions were counterbalanced within fMRI sessions, across participants. Free recall (i.e., total number of words from the SR list correctly recalled) were assessed offline, immediately after each fMRI session. Semantic clustering index scores were defined as the consecutive recall of two words from the same category. They reflected the proportion of clustered responses out of the total possible clusters defined as follows: clusters/(words recalled-categories recalled). The serial clustering score was defined as follows: clusters/(words recalled–1). Recognition was also assessed outside the scanner. Stimulus presentation and response recording were performed with E-Prime 1.0 software (Psychology Software Tools). Visual stimuli presentation was projected through a magnetic shielded glass window to a screen inside the scanner room and was synchronized with image acquisition.

### Scanning

The fMRI acquisition was based on T2^*^-weighted echo planar (EPI GRE) images for the whole brain acquired in a 3 Tesla Philips Achieva system with an eight-channel head coil. The acquisitions parameters were: TR = 3000 ms, TE = 30 ms, 40 slices, 3 mm slice thickness, 0.3 mm slice gap, FOV = 240 mm^2^ and matrix 64 × 64, 3 mm^3^ voxels, with 94 volumes per run. Functional acquisitions were preceded by four dummy scans to ensure steady-state magnetization. A T1-weighted structural image (voxel size: 1 mm^3^) was acquired before the functional sessions for coregistration with the fMRI data and to exclude brain pathology. In particular, white matter lesions were analyzed according to the Fazekas Score (Fazekas et al., 1987).

### Data analysis

Data processing and statistical analyses were conducted using FSL (www.fmrib.ox.ac.uk/fsl/) (Smith et al., 2004). Functional volumes were processed by movement correction (MCFLIRT), spatial smoothing (FWHM = 5 mm) and spatial normalization to standard space (affine, 12 DoF). Time-series from each voxel were high-pass filtered with a cut-off period of 1/100 Hz to remove signal drift and low-frequency noise. Statistical maps of activity at the individual level were calculated using the general linear model (GLM) using FILM routines (Woolrich et al., 2004), which is based on semi-parametric estimation of residuals autocorrelation. Each block (SR and UR) was modeled using a boxcar function convolved with a gamma-derived hemodynamic response function (standard deviation of 3 s, mean lag of 6 s), and the contrasts SR > fixation and UR > fixation were estimated for each participant. In a preliminary analysis, these contrasts were then entered into a second-level analysis to test if there were a main effect of list (SR, UR) or a list^*^session (spontaneous, directed) interaction, in order to identify regions whose activation increased or decreased between sessions, more for the SR contrast than the UR contrast. There were no brain regions that showed a significant interaction or a main effect of list. The remaining analyses focused only in the contrast SR > fixation, giving the sensitivity of the SR word list in prompting measurable semantic clustering scores.

Differences between the two encoding conditions were initially examined in each group separately. The effect of the explicit orientation to apply the semantic learning strategy in activation (directed > spontaneous) and deactivation (spontaneous > directed) between sessions were identified using a paired *t*-test. To answer the primary aim of this study (i.e., understanding the contribution of differences in verbal learning strategic processing between normal controls and MCI during episodic memory encoding), the interaction group (MCI, control)^*^session (spontaneous, directed) was examined, using free recall score as a covariate to control for the possibility that activation differences between groups could reflect only the performance differences between groups. To further explore the relation between differences in session-related changes in activation and strategic performance behavior, the change in semantic clustering index for each participant (directed—spontaneous, Δ strategy session) was regressed on the corresponding change in BOLD activation (Δ BOLD session). For this, a map was created in a second-level analysis subtracting the map associated with the encoding of the SR list at the spontaneous session from the directed session for each participant. To verify whether there was a difference in brain-behavior correlation patterns between groups, the interaction group^*^Δ strategy session was examined. All the statistical images were thresholded by using Gaussian random field-based cluster inference with a threshold of *Z* > 2.3 at the voxel level and a corrected cluster significance threshold of *P* < 0.05.

## Results

### Neuropsychological and behavioral data

Table 1 shows participants demographic and neuropsychological characteristics. There were no differences in age, years of formal education, socioeconomic status, and Fazekas score between groups. Subjects with MCI showed, as expected, a pattern of mild neuropsychological deficits relative to controls, particularly in the domain of memory. There were statistically significant differences in the mean scores between groups in the word list (RAVLT) and prose passages recall (WMSR), visual memory recall (Rey Figure), semantic and phonemic verbal fluency tests (Mattis), in the forward and backward digit span subtest (WAIS III), and in the Boston naming test. The groups were matched on qualitative measures of cerebrovascular integrity (e.g., white matter hyperintensities).

Behavioral performance in the word list learning paradigm related to the spontaneous and the directed fMRI sessions (Table 2) was analysed with two (session) × two (group) × two (list) repeated measures ANOVA (Table 2). A significant session effect was observed, [*F*_(1, 33)_ = 20.766, *p* < 0.001], as both groups improved their free recall from the spontaneous to the directed session, after instructions to apply the semantic encoding strategy. There was also a significant list effect [*F*_(1, 33)_ = 115.447, *p* < 0.001], showing better performance for the SR than the UR list. Controls recalled more words than MCI, independently of the list type and session [*F*_(1, 33)_ = 13.923, *p* = 0.001]. *Post-hoc* analysis indicated that MCI free recall performance after the orientation session to apply the semantic encoding strategy became similar to the controls performance at the spontaneous session [*t*_(33)_ = −1.18, *p* = 0.272].

**Table 2:** **Free recall and semantic clustering performance relative to the spontaneous and the directed sessions**.

	**Controls**		**MCI**	
	**Spontaneous**	**Directed**	**Spontaneous**	**Directed**
**FREE RECALL**				
SR	7.53 (0.575)	9.41 (0.810)	4.77 (0.583)	6.38 (0.813)
UR	3.58 (0.496)	3.29 (0.496)	1.33 (0.489)	2.278 (0.424)
Clustering index	0.242 (0.053)	0.452 (0.052)	0.182 (0.051)	0.447 (0.050)
Number of clusters	3.23 (0.390)	4.58 (0.575)	1.55 (0.379)	3.77 (0.537)

*Results are expressed as mean (SE)*.

A two (group) × two (session) repeated measures ANOVA on the semantic clustering score revealed a significant session effect [*F*_(1, 33)_ = 28.859, *p* < 0.001], indicating that both groups recalled a great number of clustered words after the explicit orientation to apply the verbal learning strategy than using self-initiated encoding strategies. The mean number of clusters generated by MCI patients was lower than the performed by controls in both sessions [*F*_(1, 33)_ = 4.275, *p* = 0.047], as expected from their lower recall performances relative to controls. Mean recognition scores indicated above chance level of performance for both groups (Supplementary Material Table S1).

### fMRI results

#### Brain activation related to changes in the application of the semantic encoding strategy across sessions—within-group comparisons

**Figure 1:**
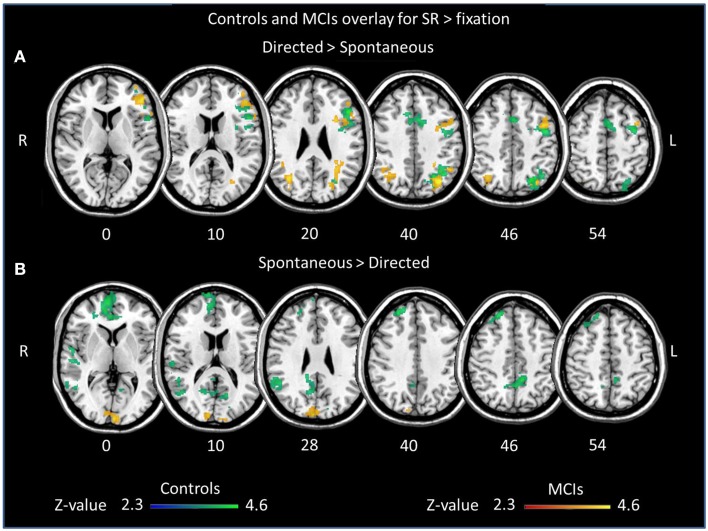
**Overlay of clusters exhibiting significant differences in activation between sessions during word list encoding for controls (blue/green) and MCIs (red/yellow)**. Brain regions showing greater **(A)** activation and **(B)** deactivation in the directed session, during the explicit application of the semantic strategy, in comparison to the spontaneous encoding session.

We first assessed differences in verbal learning strategy application across sessions in fMRI activation and deactivation during episodic encoding of word lists separately for each group (Table 3, Figure 1). Increased activation during encoding after the explicit orientation to apply the verbal organizational learning strategy (directed > spontaneous) were observed in both the MCI and control group in clusters encompassing portions of the left middle frontal gyrus (midDLPFC), inferior frontal gyrus (VLPFC) dorsal premotor cortex, and posterior parietal cortex (PPC), in the angular gyrus and within intraparietal sulcus (IPS) borders. Decreased activation (directed < spontaneous, Supplementary Material Figure S1) was observed in a set of clusters within the right superior frontal gyrus, the vmPFC, left inferior parietal cortex and infero-lateral temporal cortex, and posterior cingulate/precuneous cortex only in the control group. In the MCI group, deactivation was observed only in a small cluster located in the parieto-occipital cortex.

**Table 3:** **Statistical information of significant clusters highlighted when comparing the BOLD response between the spontaneous and directed use of learning strategies during word list encoding compared to fixation baseline**.

**Contrast**	**Region**	**Side**	**MNI coordinates**				**Size**	**Cluster *p*-values**
			***x***	***y***	***z***			
Directed > spontaneous								
controls	VLPFC						2131	< 0.0001
	Inferior frontal gyrus (BA 44)	L	−42	10	32	4.6		
	Inferior frontal gyrus (BA 45)	L	−46	24	24	4.25		
	midDLPFC						1021	< 0.0001
	Middle frontal gyrus (BA 46)	L	−36	0	54	4.04		
	Precentral gyrus (BA 6)	L	−42	0	32	4.17		
	Posterior parietal cortex						977	< 0.0001
	Intraparietal sulcus (BA 7)	L	−24	−70	48	4.27		
	Angular gyrus (BA 40)	L	−34	−58	40	3.58		
	Pre−SMA						545	0.0032
	Superior frontal gyrus (BA 6)	L	−6	2	58	4.66		
	Cingulate gyrus (BA 32)	R	6	20	36	3.86		
	Cerebelum	L	−40	−76	−26	4.27	412	0.0021
MCI	DLPFC						1416	< 0.0001
	Middle frontal gyrus (BA 9)	L	−42	8	50	3.95		
	Middle frontal gyrus (BA 46)	L	−44	44	12	3.82		
	Inferior frontal gyrus (BA 47)	L	−46	38	6	3.44		
	Inferior frontal gyrus (BA 47)	L	−52	18	0	3.71		
	Left temporoparietal cortex						1003	< 0.0001
	Intraparietal sulcus superior	L	−28	−62	32	4.56		
	Angular gyrus (BA 39)	L	−34	−70	38	4.45		
	Superior temporal gyrus (BA 22)	L	−42	−50	22	3.27		
	Right temporo−parietal						654	0.00015
	Supramarginal gyrus (BA 40)	R	42	−52	32	3.51		
	Middle temporal gyrus (BA 39)	R	40	−62	32	3.48		
	Precuneus(BA 7)	R	32	−62	42	3.44		
Directed < spontaneous								
controls	Precuneus/Posterior cingulate						1894	< 0.0001
	Posterior cingulate (BA 30)	L	−16	−66	12	3.97		
	Precuneus (BA 7)	L	0	−54	46	3.83		
	mPFC						1710	< 0.0001
	Superior frontal gyrus (BA 10)	R	12	54	−4	4.37		
	Anterior cingulate gyrus (BA 24)	R	0	36	8	3.77		
	Inferior temporal						896	< 0.0001
	Inferior parietal lobule (BA 39)	R	50	−48	24	4.16		
	Supramarginal gyrus (BA 40)	R	60	−50	30	3.69		
	Middle temporal gyrus (BA 21)	R	52	−56	6	3.34		
	Superior temporal gyrus (BA 22)	R	52	−44	10	3.26		
	Superior temporal						619	0.0012
	Middle temporal gyrus (BA 21)	R	64	−6	−16	3.74		
	Superior temporal gyrus (BA 22)	R	56	−26	8	3.72		
	Superior frontal						528	0.004
	Superior frontal gyrus (BA 9)	R	24	40	46	3.77		
	Middle frontal gyrus (BA 46)	R	26	46	30	3.35		
	OFC						421	0.0171
	Middle orbital gyrus (BA11)	R	18	16	−22	3.5		
	Rectus gyrus (BA 11)	L	−6	6	−22	3.1		
MCI	Parieto−occipital						710	< 0.0001
	Lyngual gyrus (BA 18)	L	−2	−100	−4	3.65		
	Cuneus (BA 17)	L	−6	−98	8	3.6		
	Cuneus (BA 17)	R	6	−92	26	3.38		

#### Different patterns of activation/deactivation related to changes in the application of the semantic encoding strategy across sessions between groups

**Figure 2:**
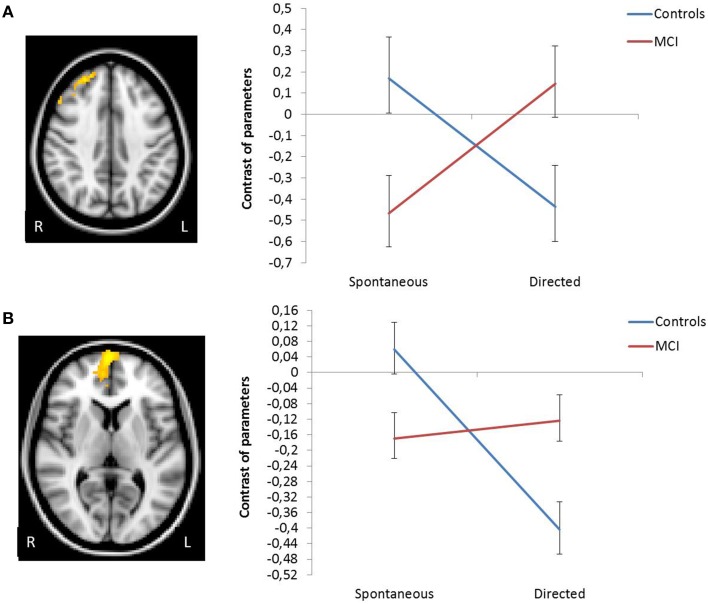
**Change in functional activation in the regions of significant group × session interaction showing that in right superior frontal gyrus (A) and in vmPFC (B) there is a decrease in activation in the controls, and an increase in MCIs**.

In order to identify brain regions that exhibited different activation/deactivation patterns during encoding with explicit instructions to apply the semantic organizational strategy relative compared to unconstraint encoding session in MCI relative to controls, the interaction group × session were examined at the whole-brain level. A significant interaction was observed in two clusters: mPFC, extending to the anterior cingulate (peak voxel MNI coordinate 2 68 8, *Z* = 4.13, cluster *p*-value corrected = 0.016), and in the right superior frontal gyrus, extending to the middle frontal gyrus (peak voxel MNI coordinate 36 32 44, *Z* = 3.46, cluster *p*-value corrected = 0.003). Plots showing the mean magnitude estimates of activity in the significant clusters for each session indicate the nature of the interaction effects, showing that only controls, but not MCI patients, exhibited a significant modulation (i.e., significant deactivation) of the mMPFC function in response to the explicit orientation to apply the encoding strategy. In the right superior frontal gyrus, group level differences followed a “cross-over” pattern, such that in controls, activation decreased after the guided use of the semantic clustering, while patients showed increased activation. The interaction remained similar after controlling for behavioral differences in word recall between groups (Figure 2).

#### Relationship between changes in strategy-based verbal learning behavior and brain activation

**Figure 3:**
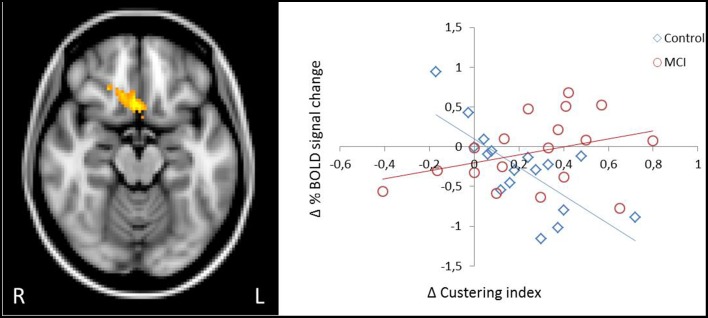
**Different patterns of association between change in semantic clustering and change in activation due to the explicit application of the encoding strategy in the OFC**. In controls, greater increase in strategic behavior was predictive of greater decrease in activation in the OFC, whereas in MCI increase in behavior was predictive of increased activation.

Given the observed group-related differences associated with the directed use of the encoding strategy in brain activation and deactivation in core regions of the cognitive control and the default-mode networks during the word list encoding task, we examined if individual changes in the fMRI BOLD signal between sessions (directed—spontaneous) in these regions would be differently associated with changes in strategic performance in controls and MCIs. A significant group by strategy interaction was observed in a cluster located in the OFC, extending to the mPFC and anterior cingulate (peak voxel MNI coordinate 4 18–16 in the rectus gyrus; *z* = 3.58; cluster *p*-value corrected = 0.0429). Scatter plots of changes in BOLD signal between sessions against changes in strategy use for this region revealed a strong negative correlation in controls, such that higher performer participants exhibited the greatest decrease in activation (*r* = −0.734), whereas in MCIs, higher performer participants exhibited the greatest increase in activation from the spontaneous to the directed session (*r* = 0.339) (Figure 3).

## Discussion

In the current study, we examined differences in fMRI brain activation and deactivation related to semantic strategy application during verbal memory encoding in MCI and HC subjects. As expected from prior reports on episodic memory performance in subjects at risk for dementia (Ribeiro et al., 2007; Hudon et al., 2011), free recall scores of the MCI group were worse than that of the control group when using self-initiated encoding strategies. However, the explicit guidance to apply the semantic strategy improved the verbal memory performance of the MCI group to the same level exhibited by control subjects when using self-initiated strategies. Improvements in verbal learning due to the application of the strategy were able to reverse MCI recall deficits to baseline controls level, which is consistent with previous findings showing that MCI can benefit from environmental support or cognitive training to reduce memory impairment (Simon et al., 2012).

Results from the fMRI analysis revealed that after the explicit orientation to apply the verbal learning strategy, greater recruitment of frontoparietal network regions, including portions of the left DLPFC, VLPFC, were observed in both MCI and control groups in relation to the unconstrained encoding condition, as expected. The initial prediction of over-recruitment of portions of the DLPFC and within the IPS in MCI reflecting possible compensation mechanisms due to increased cognitive demand to perform the task was not confirmed. Group-differences in functional deactivations, however, were observed in the vmPFC and in the right superior frontal gyrus, related to the absence of modulation in the activity of the vmPFC, along with a lack of suppression of the right superior frontal gyrus in MCI. A different association between improvement in strategy use and session-related changes in activation of the medial OFC between groups was also confirmed. As previously stated (Savage et al., 2001), participants' increased use of semantic organization strategies due to explicit orientation allowed them to monitor, update and manipulate the studied words as they mentally regrouped related words together to subsequent recall them. The increased recruitment of regions of the frontoparietal network in response to increases in strategy use during intentional episodic encoding observed in our study is consistent with findings from previous intervention studies examining the effects of memory strategy training protocols in brain activation in MCI and controls during encoding and retrieval of word lists and face-name associations (Belleville et al., 2011; Hampstead et al., 2011).

In fMRI studies, increases in activation with the development of a new strategy acquired by learning are often thought to reflect recruitment of additional cortical units, seen as strengthening of BOLD response within brain regions (Poldrack, 2000). In this context, increased activation of the frontoparietal network can be considered as an evidence of redistribution or functional reorganization of brain activation (Kelly and Garavan, 2005; Bor and Owen, 2007). However, it has also been proposed that practice improvements in applying previously learned strategies that involve organization and “chunking” (i.e., organization of small pieces of information together) also results in increased frontoparietal activation, even when task demands decrease while using these strategies (Bor and Owen, 2007). In light of these proposals, the cognitive mechanisms underlying the observed increases in frontoparietal recruitment with increases in strategy use in our study might reflect a combination of greater skill in applying a previously learned strategy, particularly in controls, and the engagement of a new strategy, especially in MCI.

Improvements in strategy application and memory performance were represented by different activation changes in the mPFC and in the right superior frontal gyrus responses between groups. These activations did not appear to be exclusively related to performance differences between groups, as we controlled for free recall scores in our fMRI analyses.

While controls clearly suppressed the responses of mPFC and of the right superior frontal gyrus during encoding after the explicit orientation to apply the semantic strategy, MCI subjects exhibited a pattern of less deactivation in these regions. Similar results were reported by the memory training study above cited (Belleville et al., 2011), but they did not directly compared the amount of training-related deactivations between MCI and controls. In the context of task induced deactivation studies, these two regions, along with the posterior cingulate and medial parietal regions and the inferior parietal lobule, have been consistently reported as nodes of the DMN of the brain (Toro et al., 2008). Although the DMN was originally proposed as a set of regions that exhibited greater blood flow or BOLD signal during baseline rest or fixation conditions than during task performance, subsequent studies demonstrated similar patterns of results when tasks with different cognitive demands were contrasted (Raichle et al., 2001). Recent studies have shown, for example, that mPFC activity is greater during performance of a relatively easy 0-back task than a 2-back working memory task (Leech et al., 2011), and that suppressing activity in DMN regions during working memory encoding was predictive of subsequent better performance (Anticevic et al., 2010). In our study, when an increased working memory demand was imposed by the direct application of the encoding strategy, controls exhibited a suppression of these regions (i.e., less activation), but not the MCI patients. Similar findings were recently reported in MCI during a graded working memory paradigm (Papma et al., 2014). It has been suggested that DMN deactivation is progressively disrupted along the continuum from normal aging to MCI and AD, with increased impairment in subjects at risk for AD, such as *APOE4* genotype carriers (Pihlajamäki and Sperling, 2009). In this context, the pattern of results in the present study suggests that MCI may be less efficient than controls in processing external task-irrelevant information during encoding, suffering in a great extent the effects of distraction.

An alternative interpretation for the different pattern of results between our groups came from evidence suggesting that when performance of a task improves after learning or practice due to better application of strategies, task becomes less effortful and demands on executive control processes are reduced (Jonides, 2004). This reduction in executive control can lead to a reduction of activation that is correlated with better performance (Poldrack, 2000). In the present study, greater reductions in activation on a region encompassing the OFC, extending to the mPFC and the anterior cingulate were strongly predicted by greater increases in strategic performance in controls, supporting a possible automaticity hypothesis.

The results of our study should be interpreted in the context of some potential limitations. The MCI diagnosis was performed based on cross-sectional neuropsychological data rather than on longitudinal subject-by-subject objective evidence of progressive memory loss, and so it is not possible to completely rule out the possibility that patients defined on the basis of their clinical profile belong to different subgroups and different points of a severity continuum. It must also be acknowledged that the ability to discriminate changes in activity that represent differences in cognitive function from those that represent physiological change due to a MCI diagnosis is a challenge for fMRI research, especially since neurovascular coupling processes may change with age or disease risk (D'Esposito et al., 2003). We attempted to decrease the possibility of confounding compromised vascular responses with changes in cognitive processing by excluding individuals with cerebrovascular disease and matching groups on qualitative measures of cerebrovascular integrity (e.g., white matter hyperintensities). Moreover, although we observed improvement in recall performance after the directed strategy application in MCI, it is possible that the relative limited stimulus exposure time (e.g., one word per 2 s) may have constraint the use of spontaneous mnemonic strategies by these participants (Hampstead et al., 2014). Present findings also require further replication in the context of an independent control group to ensure that the observed brain changes associated with the greater use of the encoding strategy are not solely due to repetition effects.

In sum, the results of our study extend the existing literature on differential cognitive processing and neural recruitment in MCI during performance on episodic memory encoding tasks. The novel contributions of our study involved an assessment of the neural correlates of strategic processes employed during encoding of episodic memory in this population. Examining differences in the patterns of deactivation during verbal encoding with increased strategic processing, we found that MCI patients failed to show modulation of activation in mPFC and less deactivation in the right superior frontal gyrus compared to normal controls. Such different pattern of responses may reflect changes in the set of cognitive processes, particularly executive functions, adopted during verbal memory encoding between normal aging and MCI.

### Conflict of interest statement

The authors declare that the research was conducted in the absence of any commercial or financial relationships that could be construed as a potential conflict of interest.

## Supplementary material

Figure S1 **Mean contrast of parameter estimates (SR > Rest) and standard errors for the comparison Directed < Spontaneous in the control group in clusters encompassing the precuneus, mPFC, and inferior temporal cortex**. The MCI group exhibited significant deactivation between sessions only in the parietal-occipital cluster (indicated with an asterisk).

Table S1 **Recognition performance on the fMRI paradigm**. Results are expressed as mean (SE).
