# Supplementary figures and images for: Differences in prefrontal cortex activation and deactivation during strategic episodic verbal memory encoding in mild cognitive impairment
Source: Front Aging Neurosci. 2015 Aug 4;7:147. doi: 10.3389/fnagi.2015.00147 (PMC4523841; doi:10.3389/fnagi.2015.00147)

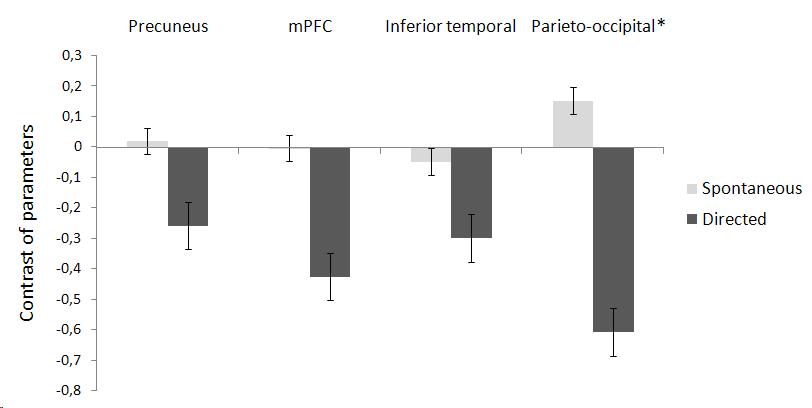

Supplement: Figure S1 — Mean contrast of parameter estimates (SR > Rest) and standard errors for the comparison Directed < Spontaneous in the control group in clusters encompassing the precuneus, mPFC, and inferior temporal cortex. The MCI group exhibited significant deactivation between sessions only in the parietal-occipital cluster (indicated with an asterisk). [file Image1.JPEG]
