# Supplementary material for: Differences in prefrontal cortex activation and deactivation during strategic episodic verbal memory encoding in mild cognitive impairment
Source: Front Aging Neurosci. 2015 Aug 4;7:147. doi: 10.3389/fnagi.2015.00147 (PMC4523841; doi:10.3389/fnagi.2015.00147)
Supplement: Table S1 — Recognition performance on the fMRI paradigm. Results are expressed as mean (SE). [file Table1.DOCX]

SUPPLEMENTARY MATERIAL

Table S1. Recognition performance on the fMRI paradigm. Results are expressed as mean (SE).

|  | Controls | | MCI | |  |  |
| --- | --- | --- | --- | --- | --- | --- |
|  | Spontaneous | Directed | Spontaneous | Directed | | |
| Recognition |  |  |  |  | | |
| Hit rate SR | 0.92 (0.022) | 0.82 (0.036) | 0.72 (0.038) | 0.70 (0.038) | | |
| Hit rate UR | 0.83 (0.036) | 0.87 (0.026) | 0.83 (0.023) | 0.79 (0.027) | | |
| Corrected rejection rate | 0.92 (0.029) | 0.89 (0.023) | 0.82 (0.031) | 0.92 (0.024) | | |
